# Species, Natural *Leishmania* spp. Detection and Blood Meal Sources of Phlebotomine Sandflies (Diptera: Psychodidae: Phlebotominae) in Peridomiciles from a Leishmaniases Endemic Area of Brazil

**DOI:** 10.1155/2024/9932530

**Published:** 2024-02-22

**Authors:** João Augusto Franco Leonel, Geovanna Vioti, Maria Luana Alves, Julio Cesar Pereira Spada, Alex Kazuo Yamaguchi, Nuno Wolfgang Balbini Pereira, Diogo Tiago da Silva, Julia Cristina Benassi, Fredy Galvis-Ovallos, Eunice Aparecida Bianchi Galati, Wilma Aparecida Starke-Buzetti, Rodrigo Martins Soares, Heidge Fukumasu, Trícia Maria Ferreira de Sousa Oliveira

**Affiliations:** ^1^Laboratory of Applied Preventive Veterinary Medicine, Department of Veterinary Medicine, Faculty of Animal Science and Food Engineering, University of São Paulo–USP, Pirassununga, State of São Paulo, Brazil; ^2^Post-Graduate Program in Experimental Epidemiology Applied to Zoonoses, Department of Preventive Veterinary Medicine and Animal Health, Faculty of Veterinary Medicine and Animal Science, University of São Paulo–USP, Pirassununga, State of São Paulo, Brazil; ^3^Laboratory of Entomology in Public Health, Department of Epidemiology, School of Public Health, University of São Paulo–USP, São Paulo, State of São Paulo, Brazil; ^4^Department of Biology and Animal Science, School of Engineering, São Paulo State University–UNESP, Ilha Solteira, State of São Paulo, Brazil; ^5^Department of Preventive Veterinary Medicine and Animal Health, Faculty of Veterinary Medicine and Animal Science, University of São Paulo–USP, Pirassununga, State of São Paulo, Brazil; ^6^Laboratory of Comparative Oncology and Translational, Department of Veterinary Medicine, Faculty of Animal Science and Food Engineering, University of São Paulo–USP, Pirassununga, State of São Paulo, Brazil

## Abstract

Leishmaniases are important neglected tropical diseases caused by protozoans of the genus *Leishmania* spp. The female sandflies bite (Diptera: Psychodidae: Phlebotominae) is the main transmission form in the epidemiological chains of these diseases. Thus, studies on Phlebotominae subfamily are fundamental to improve the comprehension of the leishmaniases epidemiology, revealing relationships of these dipterans with the parasite and reservoirs. An entomological survey of Phlebotomine sandflies was carried out to investigate the fauna, *Leishmania* spp. DNA detection and blood meals sources of female sandflies captured in peridomiciles areas from a leishmaniases endemic area of São Paulo state, Brazil. A total of 637 Phlebotomine sandflies specimens were captured, and twelve species identified, some of them proven or suspected vectors of tegumentary leishmaniasis (TL) and visceral leishmaniasis (VL). *Evandromyia* (*Aldamyia*) *carmelinoi* and *Lutzomyia* (*Lutzomyia*) *longipalpis* PCR positive to *Leishmania* (*Leishmania*) *infantum* kinetoplast minicircle DNA (kDNA) were identified. Also, *Leishmania* (*Leishmania*) *amazonensis* kDNA was found in *Evandromyia* (*Aldamyia*) *lenti* and *Lu*. (*Lu*.) *longipalpis*. Regarding blood meals source, DNA of swine, humans, dogs, cattle, chickens, and opossums were detected in the midgut of Phlebotomine sandflies females captured in the study area. Our results highlight ecological relationships among different species of Phlebotomine sandflies, domestic and wild-synanthropic vertebrates (including humans) and two *Leishmania* species in peridomiciles from a leishmaniases endemic area of Brazil.

## 1. Introduction

Leishmaniases are important vector-borne diseases caused by parasites from the genus *Leishmania* and endemic in 102 countries and territories worldwide [1, 2]. Considered as neglected tropical diseases, leishmaniases are strongly linked with poverty, affecting mainly poor populations in developing countries, where they have great importance in public health [3].

A better comprehension of *Leishmania* spp. transmission cycle requires that all relationship among parasites, vectors, and vertebrate hosts reservoir to be determined and clarified [4]. The sandflies studies are driven by the fundamental role of these insects in the epidemiology of the disease [5]. Called by “sandfly” due to the pale or sandy body color, Phlebotomine sandflies (Diptera: Psychodidae: Phlebotominae) are the main vector of leishmaniases [6]. Nowadays, 1,060 sandflies species (1,028 extant and 32 fossil) have been cataloged around the world of which 555 are present in the Americas (538 extant and 17 fossil) [7]. Of the 212 sandflies species recorded in Brazil, 78 species are present in the São Paulo state [7, 8].

Studies about biology of these dipterans and their relationships among vertebrate reservoir and *Leishmania* spp. parasite constitute an important epidemiological tool [9–11]. Investigations about natural *Leishmania* spp. infection and blood meals source of females sandflies in endemic areas give an important epidemiological information [11–13]. These investigations can point out a potential vector or vertebrate host reservoir and help to improve the knowledge about the complex ecoepidemiology of leishmaniases in an area, to lead the adoption of more effective control measures [9–12, 14–18].

Over the past 21 years, approximately 1,100,000 tegumentary leishmaniasis (TL) human cases were reported in Americas, of which 37.6% occurred in Brazil [19]. In 2021, thereabout 15,000 TL human cases were notified in the country, and about 200 cases were registered in the state of São Paulo [19, 20]. On the other hand, approximately 70,000 visceral leishmaniasis (VL) human cases have been recorded in the Americas between 2001 and 2021 [19]. In this year, the *Leishmania* (*Leishmania*) *infantum* infection was reported in nearly 1,800 Brazilian citizens, and 65 residents of cities in São Paulo state [19, 21]. Herein, we performed an entomological survey of Phlebotomine sandflies to investigate the fauna, *Leishmania* spp. DNA detection and blood meals identification of female sandflies caught at peridomiciles from a leishmaniases endemic area in a northwest of São Paulo state, Brazil.

## 2. Methods

### 2.1. Study Area

The Andradina and Ilha Solteira counties are located in the administrative mesoregion of Araçatuba, São Paulo state, about 650 km of São Paulo city, capital of the state. According to the Köppen and Geiger's classification, the climate is “Aw” type, humid tropical with dry season, associated with the Cerrado biome, with annual average temperature of 24.7°C and 1,302 mm of average annual rainfall [22].

### 2.2. Phlebotomine Sandflies Samples

Between 2018 and 2020, captures of Phlebotomine sandflies were carried out with CDC light traps in seven ecotopes in urban and rural area for each county. Two CDC light traps per point were installed in the peridomicile areas, near to animal shelters or vegetation located between 10 and 20 m from the house [23]. The light traps were set up about one meter from the ground, between 17:00 and 7:00 h [23].

### 2.3. Phlebotomine Sandflies Species Identification

The insects captured were readily taken to the laboratory in a styrofoam box with ice [24]. Then, they were euthanized in a freezer at −20°C for 20 min and screened [24]. The sandflies were counted and registered according to sex, place, and date of capture. Males and females (head and terminalia) were preserved individually in sterile microtubes with alcohol 70%. Next, they were clarified and mounted on glass slides for species identification according to Galati et al. [25] and genera abbreviation according to Marcondes et al. [26]. In addition, thorax and abdomen of females was preserved individually in sterile tubes with absolute alcohol (Merck) at −20°C until DNA extraction and molecular analyses [24]. The instruments used in insects screening step and working areas were disinfected with a 2% sodium hypochlorite solution [27].

### 2.4. Molecular Analyses

#### 2.4.1. DNA Extraction

DNA extraction from thorax and abdomen of each females Phlebotomine sandflies was performed in according to Bruford et al. [28], with modification by Galvis–Ovallos et al. [23]. To avoid DNA contamination among samples, proceedings were carried out in a biological safety cabin (ESCO). The instruments and cabin were disinfected as described above, followed by 15 min in UV.

#### 2.4.2. Conventional PCR

DNA extracted from thorax and abdomen of individual females Phlebotomine sandflies were submitted to conventional PCR. In order to evaluate the quality of DNA extraction, a PCR amplification for the IVS6 region of the cacophony gene of Phlebotomine sandflies (endogenous gene control) was made with primers described by Lins et al. [29] and according to Pita–Pereira et al. [30]. To detect *Leishmania* spp. DNA, a PCR was performed to kinetoplast minicircle DNA (kDNA) of parasite as described by Volpini et al. [31]. *Leishmania* spp. kDNA positive samples were submitted to PCR by genomic region of ribosomal DNA (rDNA) using Internal Transcribed Spacer 1 and 2 genes (ITS-1 and ITS-2) according to El Tai et al. [32]. A PCR for blood meals identification was made targeting the conserved region of the cytochrome B gene (CYT-B) of vertebrate's mitochondrial DNA (mtDNA) described by Steuber et al. [33] only in DNA samples from engorged females.

All amplifications were performed in a thermocycler C1000 Touch™ thermal cycler (Bio-Rad) and sterile deionized water was used as a negative control. DNA samples extracted from *Lutzomyia* (*Lutzomyia*) *longipalpis* (Lutz and Neiva, 1912) males from the colony of Laboratory of the Entomology in Public Health/Phlebotominae (LESP) of the Faculty of Public Health (FSP), University of São Paulo (USP) were used as a positive control in PCR to endogenous gene control. While *Leishmania* (*Leishmania*) *amazonensis* (IFLA/BR/1967/ph8) provided by the Leishmaniasis Laboratory of the Oswaldo Cruz Institute (FIOCRUZ), Rio de Janeiro/RJ, was used as a positive control in PCR to *Leishmania* spp. DNA detection. In addition, DNA from dog (*Canis lupus familiaris*) was used as a positive control in the PCR to identify sandflies blood meals.

Twelve microliters of PCR products were mixed with 3 *μ*l of sample buffer (10 mM Tris, 10 mM EDTA, 0.005% *m*/*v* bromophenol blue, and 10% *v*/*v* glycerol) and subjected to electrophoresis on 1.5%–2% agarose gel stained with SYBR® Safe (Invitrogen). The run was performed in 1× TBE buffer (pH 8.0; Invitrogen) at 100 V for 45–60 min with 100 bp DNA ladder RTU® (KASVI). A UV Photo Doc-It® (UVP) transilluminator was used to view and photograph the amplified products in agarose gel.

#### 2.4.3. Restriction Fragment Length Polymorphism PCR

The PCR products from the amplification of *Leishmania* spp. kDNA were subjected to digestion with Hae III restriction enzyme (Promega) for *Leishmania* spp. identification. To this end, 20 *μ*l of the amplified product were used for digestion by the addition of 2 *μ*l of Hae III (10 U/*μ*l), 2 *μ*l 10x MULTI-CORE™ buffer, and 0.2 *μ*l of acetylated BSA (10 *μ*g/*μ*l), followed by incubation for 3 hr at 37°C [31]. Restriction fragments were separated by capillary electrophoresis gel using standard DNA cartridge kit (BiOptic) in GelBot HT (Loccus) equipment. The results were analyzed by Qsep series software v.3.3.0. The restriction fragments were compared to the reference samples of *L*. (*L*.) *amazonensis* (IFLA/BR/1967/ph8), *Leishmania* (*Viannia*) *braziliensis* (MCAN/BR/1987/CÃO21) and *L*. (*L*.) *infantum* (MCAN/BR/1984/CCC-17.481) all provide by Leishmaniasis laboratory of FIOCRUZ/Rio de Janeiro/RJ.

#### 2.4.4. Sequencing

Positive PCR samples to ITS-1, ITS-2, and CYT-B genes were sent for Sanger sequencing using 30 ng/*µ*l of purified PCR product. The software Chromas was used to manually check the electropherograms from forward and reverse sequence. The BioEdit sequence alignment editor was used to align and generate consensus sequence. After, these sequences were confronted for regions of similarity with sequences from GenBank by Basic Local Alignment Search Tool (BLAST). Species identification of *Leishmania* spp. and vertebrates were considered correct when the sequences showed over 95% identity for 100% of the analyzed sequence.

## 3. Results

### 3.1. Phlebotomine Sandflies Fauna

The captures revealed 94 specimens of the Phlebotomine sandflies in Andradina, of which 54 were males and 40 females (Table 1). In addition, 543 specimens were captured in Ilha Solteira, 316 males and 227 females (Table 2).

**Figure 1:**
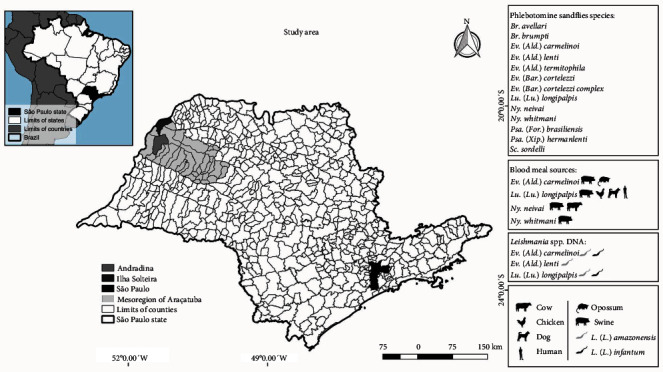
Phlebotomine sandflies species, *Leishmania* spp. DNA detection and blood meals sources on captured-females sandflies at peridomiciles from the northwest of São Paulo state, Brazil. Footnote: Map made with the QGIS 2.18 “Las Palmas” software, using free access datasets from Instituto Brasileiro de Geografia e Estatística (IBGE) and free icon vectors from vecteezy.com and flaticon.com.

Twelve Phlebotomine sandflies species were found and distributed into three subtribes: BRUMPTOMYIINA – *Brumptomyia avellari* (Costa Lima, 1932) and *Brumptomyia brumpti* (Larrousse, 1920); LUTZOMYIINA – *Evandromyia* (*Aldamyia*) *carmelinoi* (Ryan, Fraiha, Lainson and Shaw, 1986), *Evandromyia* (*Aldamyia*) *lenti* (Mangabeira, 1938), *Evandromyia* (*Aldamyia*) *termitophila* (Martins, Falcão and Silva, 1964), *Evandromyia* (*Barretomyia*) *cortelezzii* (Brèthes, 1923), *Lu*. (*Lu*.) *longipalpis* (Lutz and Neivai, 1912), and *Sciopemyia sordellii* (Shannon and Del Ponte, 1927); and PSYCHODOPYGINA – *Nyssomyia neivai* (Pinto, 1926), *Nyssomyia whitmani* (Antunes and Coutinho, 1939), *Psathyromyia* (*Forattiniella*) *brasiliensis* (Costa Lima, 1932), *Psathyromyia* (*Xiphopsathyromyia*) *hermanlenti* (Martins, Silva, and Falcão, 1970; Tables 1 and 2; Figure 1).

Seven species were found in residences from both counties, and five appeared only in Ilha Solteira (Tables 1 and 2). Nineteen specimens were damaged during handling and due to this; species-level identification was impaired. Thus, these insects were identified only at subtribe level, LUTZOMYIINA (six specimens) and genera level, *Brumptomyia* spp. (six specimens), *Evandromyia* spp. (three specimens), and *Nyssomyia* spp. (four specimens; Tables 1 and 2). Moreover, it was not possible to define the identification between *Ev*. (*Bar*.) *cortelezzii* or *Evandromyia* (*Barretomyia*) *sallesi*, due to the absence of the male from these species in the same ecotopes (AND-3 and IS-7) [34]. Thus, these specimens were considered as *Ev*. (*Bar*.) *cortelezzii* complex (Tables 1 and 2), leading to highlight of the possible occurrence of *Ev*. (*Bar*.) *sallesi* in the study area.

### 3.2. Molecular Analyses

#### 3.2.1. Endogenous Gene Control

Two hundred sixty-seven female Phlebotomine sandflies samples were subject to IVS6 cacophony gene PCR amplification to confirm DNA extraction. Three samples (from Ilha Solteira) were negative and excluded from subsequential analysis. Thus, a total of 264 individual female Phlebotomine sandflies were screened by *Leishmania* spp. DNA.

#### 3.2.2. Phlebotomine Sandflies Positive by *Leishmania* spp. DNA

The PCR target to *Leishmania* spp. kDNA showed, one *Ev*. (*Ald*.) *carmelinoi* positive for *Leishmania* spp. kDNA, that means a 2.50% (1/40) of natural positive PCR rate on females from Andradina (Figure 1). Regarding Ilha Solteira, one *Ev*. (*Ald*.) *lenti* and three *Lu*. (*Lu*.) *longipalpis* were positive and so a 1.79% (4/224) of natural females' positive PCR rate was estimated to the county (Figure 1).

The PCR target for rDNA using the ITS-1 and ITS-2 genes showed amplification for only one *Lu*. (*Lu*.) *longipalpis* DNA sample from Ilha Solteira. The sequencing of both genes showed 100% similarity with *L*. (*L*.) *infantum*. The ITS-1 and ITS-2 sequences were deposited on GenBank under accession number OQ944444 and OQ944465, respectively.

#### 3.2.3. kDNA *Leishmania* spp. RFLP-PCR

**Figure 2:**
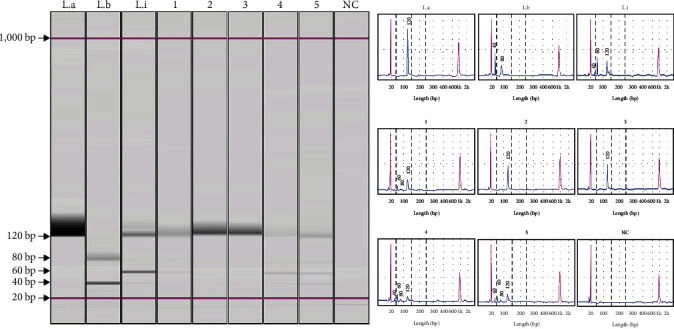
RFLP-PCR products from the *Leishmania* spp. kDNA digested with Hae III restriction enzyme in captured-females Phlebotomine sandflies DNA samples from Andradina and Ilha Solteira county, São Paulo state by CDC light traps between 2018 and 2020. Footnote: Capillary electrophoresis gel for kDNA *Leishmania* spp. PCR products after digestion by Hae III. DNA samples of *L*. (*L*.) *amazonensis* reference strain (IFLA/BR/1967/ph8) (L.a), *L*. (*V*.) *braziliensis* reference strain (MCAN/BR/1987/CÃO21) (L.b), *L*. (*L*.) *infantum* reference strain (MCAN/BR/1984/CCC-17.481) (L.i), *Ev*. (*Ald*.) *carmelinoi* female (1), *Ev*. (*Ald*.) *lenti* female (2), *Lu*. (*Lu*.) *longipalpis* female (3–5), and ultrapure water (negative control–NC).

The Phlebotomine sandflies PCR positive for *Leishmania* spp. kDNA were submitted to RFLP-PCR to *Leishmania* spp. identification. The *Leishmania* spp. kDNA digestion by HAE III showed one *Ev*. (*Ald*.) *carmelinoi* and two *Lu*. (*Lu*.) *longipalpis* PCR positive to *L*. (*L*.) *infantum* kDNA (Figure 2) and *L* (*L*.) *amazonensis* kDNA was found in one *Ev*. (*Ald*.) *lenti* and one *Lu*. (*Lu*.) *longipalpis* (Figure 2).

#### 3.2.4. Vertebrate's Blood Source for Female Phlebotomine Sandflies

Blood meals identification was performed by PCR target to CYT-B gene of vertebrate's mtDNA on DNA samples from 12 engorged female Phlebotomine sandflies, followed by sequencing. Genetic sequencing of a fragment encoding a 359 bp showed female sandflies blood positive for six vertebrate species: *Sus scrofa* (6/12; 50.00%), *Homo sapiens* (2/12; 16.67%), *C. lupus familiaris* (1/12; 8.33%), *Bos taurus* (1/12; 8.33%), *Gallus gallus* (1/12; 8.33%), and *Didelphis albiventris* (1/12; 8.33%; Figure 1). The CYT-B sequences were deposited on GenBank under accession numbers OQ982475, OQ982476, OQ982477, OQ982478, OQ982479, OQ982480, OQ982481, OQ982482, OQ982483, OQ982484, OQ982485, and OQ982486.

## 4. Discussion

Herein the Phlebotomine sandflies species, *Leishmania* spp., and vertebrate's blood DNA on captured-females sandflies were investigated at peridomiciles in a TL and VL endemic area from the northwest of São Paulo state in Brazil (Figure 1). This is an important region considered as the “front door” of VL in state, registering the first human and canine cases [35].

A total of 637 specimens of the Phlebotomine sandflies were captured, being 58.08% (370/637) males and 41.91% (267/637) females. Twelve sandflies species were found, two of them were the main responsible for *L*. (*L*.) *infantum* and *L*. (*V*.) *braziliensis* transmission in Brazil, as *Lu*. (*Lu*.) *longipalpis* and *Ny*. *whitmani*, respectively [36, 37]. The *Lu*. (*Lu*.) *longipalpis* was the major sandfly species caught in almost all ecotopes whether in peridomiciles of urban or rural area from both cities. In fact, *Lu. (Lu*.) *longipalpis* appears to be completely adapted to the anthropic environment, as already observed in other areas [27, 38]. Despite the presence of TL and VL proven vectors, some other species found also deserve attention; *Ev*. (*Ald*.) *lenti*, *Ev*. (*Bar*.) *cortelezzii*, *Ev*. (*Bar*.) *sallesi*, *Ev*. (*Bar*.) *cortelezzii* complex, *Ny. neivai* and *Sc. sordelli* has been described infected by *L*. (*V*.) *braziliensis* and *L*. (*L*.) *infantum* [4, 39, 40].

A DNA-banking samples of thorax and abdomen from 264 wild-captured female Phlebotomine sandflies were submitted to *Leishmania* spp. kDNA detection. This PCR has been considered very sensitive to DNA detection in sandflies with threshold detection of 0.004 parasites [41]. Thus, considering females sandflies captured from both counties, we found 1.89% (5/264) of female sandflies *Leishmania* spp. kDNA positive by PCR. Studies investigating the presence of *Leishmania* spp. in sandflies are fundamental in epidemiological studies of leishmaniases, providing information that helps to elucidate the patterns and intensity of transmission in an area [12, 42, 43]. The natural infection rate has been traditionally estimated, with low sensitivity, by sandflies females laborious dissection under the microscopic and *Leishmania* protozoa visualization in their gut [30, 41]. In this sense, molecular methods, such as PCR, are being used to determine the sandflies rate infection [41, 42].

We found *Leishmania* spp. kDNA in an *Ev*. (*Ald*.) *carmelinoi* female from a peridomicile of rural area from Andradina. Also, one *Ev*. (*Ald*.) *lenti* and three *Lu*. (*Lu*.) *longipalpis* from a peridomestic areas of rural area from Ilha Solteira were *Leishmania* spp. kDNA PCR positive. The *Leishmania* spp. PCR with rDNA target using ITS-1 and ITS-2 regions, was used for identification of parasite species by sequencing. The sequencing of these regions was able to identify a *L*. (*L*.) *infantum* rDNA only in one *Lu*. (*Lu*.) *longipalpis* positive sample.

Furthermore, the *Leishmania* spp. species identification was also performed by the kDNA *Leishmania* spp. RFLP-PCR. The *Leishmania* spp. kDNA digestion by HAE III showed patterns that allowed to identify *L*. (*L*.) *infantum* kDNA in one *Ev*. (*Ald*.) *carmelinoi* and two *Lu*. (*Lu*.) *longipalpis*. In addition, *L*. (*L*.) *amazonensis* kDNA was found in one *Ev*. (*Ald*.) *lenti* and one *Lu*. (*Lu*.) *longipalpis*.

Although *Lu*. (*Lu*.) *longipalpis* is a confirmed *L*. (*L*.) *infantum* vector, this sandfly species is also considered a permissive vector, since it is susceptible to infection by others *Leishmania* species, including *L*. (*L*.) *amazonensis* [38, 44, 45]. In this line, the permissivity of *Ev*. (*Ald*.) *lenti* was suggested by Lana et al. [40] that reported *L*. (*L*.) *infantum* and *L*. (*V*.) *braziliensis* DNA in females of this species. Also reported positive to *L*. (*L*.) *amazonensis* DNA in this study.

As far as we know, this is the first report of *Ev*. (*Ald*.) *carmelinoi* and *Ev*. (*Ald*.) *lenti* PCR positive for *L*. (*L*.) *infantum* and *L*. (*L*.) *amazonensis* kDNA, respectively. The simple *Leishmania* spp. kDNA findings on these specimens does not imply that these species might play a role in leishmaniases transmission [40]. Experimental infections and vector competence studies are required to elucidate the relationship between parasite and these sandflies species [4, 40, 46].

Blood feeding identification was identified in 12 engorged female Phlebotomine sandflies of four species captured: *Ev*. (*Ald*.) *carmelinoi*, *Lu*. (*Lu*.) *longipalpis*, *Ny*. *neivai*, *and Ny*. *whitmani*. We found female sandflies blood feeding on six vertebrate species. Swine (*S. scrofa*) were the major blood meal (50.00%) followed by humans (*H. sapiens*) (16.67%), dogs (*C. lupus familiaris*), cattle (*B. taurus*), chickens (*G. gallus*), and opossum (*D. albiventris*; 8.33% each).

Major feeding habits on swine were also reported by other studies [11, 47, 48]. In this survey, swine were the blood source to *Ev*. (*Ald*.) *carmelinoi*, *Lu*. (*Lu*.) *longipalpis*, *Ny*. *neivai*, and *Ny*. *whitmani* (Figure 1). Even at low frequency, chickens also were blood source to *Lu*. (*Lu*.) *longipalpis* (Figure 1). Although chickens are resistant to *Leishmania* infection, they suffer frequently blood repast by *Lu*. (*Lu*.) *longipalpis* [49]. These findings reinforce the importance of swine and chickens in peridomicile area, serving as a blood source by female Phlebotomine sandflies. In this way, these vertebrate species can attract the vectors to peridomicile, contributing to increase the risk of *Leishmania* spp. infection to the neighborhood [50]. Moreover we have found *Lu*. (*Lu*.) *longipalpis* female fed on dog (Figure 1), the main reservoir host in the urban VL cycle [51]. Also, *Lu*. (*Lu*.) *longipalpis* females showed anthropophilic feeding habits in this study (Figure 1).

One *Ev*. (*Ald*.) *carmelinoi* female was found engorged by opossum blood (Figure 1). The opossum are considered a VL potential reservoir and its presence has been incriminated such a factor associated with *L*. (*L*.) *infantum* infection in peridomicile in Brazil [52, 53]. Also, one *Ny*. *neivai* female was found fed on a bovine in this study (Figure 1). One *Lu (Lu*.) *longipalpis* female fed on cattle was reported in another endemic area of Brazil [54]. Although the role of cattle in leishmaniases epidemiology needs further studies, *L*. (*L*.) *infantum* DNA was detected in a bovine from São Paulo state in Brazil [55]. As remembered by Paternina et al. [13], the blood meal source identified in our study, do not necessarily infer a preference habit of these insects for these vertebrates. However, these findings can help to understand the Phlebotomine sandflies host's profile in the studied area [9, 18].

Although there has been a downward trend in the number of TL and VL human cases in the Americas [19], data from the São Paulo Public Health Service shows 16 VL and 5 TL human cases in the region studied on last 5 years [20, 21]. In the last years, changes in the leishmaniases epidemiological patterns have been observed in Brazil [19, 36, 37]. The TL which had a predominantly wild transmission pattern, related to occupational and leisure activities in forest environments, currently shows a pattern of transmission occuring in the peridomestic environment, reflected by the incresead of TL cases in women and children under 10 years old [19, 37]. Likewise, VL was previously considered a disease restricted to rural areas of Brazil, has spread to urban areas, with autochthonous human and canine cases reported in several urban centers of the country [19, 36].

The environmental changes caused by urbanization processes (such as deforestation, migration, irregular land occupation, poor sanitation, and increase of animals domestic in peridomestic environment) have been related to changes in the transmission patterns of leishmaniases observed [36, 37, 56, 57]. In particular, leading to significant environmental changes and favoring the adaptation of vector and reservoir species in the peridomestic environment [27, 56, 57]. In this entomological survey, we observed the presence of proven and suspected vectors in the leishmaniases transmission in peridomiciles from Andradina and Ilha Solteira municipalities. Also, interactions between the Phlebotomine sandflies fauna and VL reservoirs (dogs and opossum) were demonstrated in the study area.

In conclusion, several studies have demonstrated the dynamism of Phlebotomine sandflies population and have drawn attention to the need for continuous entomological surveillance to contain the spread of the disease [19, 58]. Our results demonstrate a network of ecological relationships that involve different species of Phlebotomine sandflies, domestic and wild-synanthropic vertebrates (including humans), and two *Leishmania* species.

## 5. Conclusion

Phlebotomine sandflies proven and suspected TL and VL vectors were captured in important counties of northwest of São Paulo state. In addition, *L*. (*L*.) *infantum* DNA and *L*. (*L*.) *amazonensis* DNA were detected in female Phlebotomine sandflies, in particular for the first time in specimens of *Ev*. (*Ald*.) *carmelinoi* and *Ev*. (*Ald*.) *lenti*, respectively. Swine, humans, dogs, cattle, chickens, and opossums were proven to have a host relationship with female Phlebotomine sandflies.

## Figures and Tables

**Table 1:** Phlebotomine sandflies species catches in peridomiciles of Andradina county, São Paulo state, between 2018 and 2020, described by capture ecotopes and sandflies sex with absolute and relative numbers.

Species/Ecotopes	Ecotope 1		Ecotope 2		Ecotope 3		Ecotope 4		Ecotope 5		Ecotope 6		Ecotope 7		Total		
	♂	♀	♂	♀	♂	♀	♂	♀	♂	♀	♂	♀	♂	♀	♂	♀	♂♀
*Brumptomyia* spp.	—	1	—	—	—	—	—	—	—	—	—	—	—	—	0	1 (1.06%)	1 (1.06%)
*Evandromyia* spp.	—	—	—	—	—	—	—	—	—	—	—	—	—	1	0	1 (1.06%)	1 (1.06%)
*Ev*. (*Ald*.) *carmelinoi*	—	—	—	1	1	1	2	2	—	1	6	11	—	3	9 (9.57%)	19 (20.21%)	28 (29.79%)
*Ev*. (*Ald*.) *lenti*	—	—	—	—	2	—	—	—	—	—	14	2	1	—	17 (18.09%)	2 (2.13%)	19 (20.21%)
*Ev*. (*Bar*.) *cortelezzii*	—	—	—	—	—	—	—	—	—	—	1	1	—	—	1 (1.06%)	1 (1.06%)	2 (2.13%)
*Ev*. (*Bar*.) *cortelezzii* complex	—	—	—	—	—	1	—	—	—	—	—	—	—	—	0	1 (1.06%)	1 (1.06%)
*Lu*. (*Lu*.) *longipalpis*	—	—	8	5	2	1	—	—	1	—	5	2	7	1	23 (24.47%)	9 (9.57%)	32 (32.04%)
*Ny. neivai*	1	—	—	—	—	—	—	—	1	1	—	1	—	—	2 (2.13%)	2 (2.13%)	4 (4.26%)
*Ny*. *whitmani*	—	—	—	—	1	—	—	—	—	—	—	1	—	—	1 (1.06%)	1 (1.06%)	2 (2.13%)
*Sc. sordellii*	—	—	—	—	—	—	—	—	—	—	1	3	—	—	1 (1.06%)	3 (3.19%)	4 (4.26%)

Total	1	1	8	6	6	3	2	2	2	2	27	21	8	5	54 (57.45%)	40 (42.55%)	94 (100%)

**Table 2:** Phlebotomine sandflies species catches in peridomiciles of Ilha Solteira county, São Paulo state, between 2018 and 2020, described by capture ecotopes and sandflies sex with absolute and relative numbers.

Species/ecotopes	Ecotope 1		Ecotope 2		Ecotope 3		Ecotope 4		Ecotope 5		Ecotope 6		Ecotope 7		Total		
	♂	♀	♂	♀	♂	♀	♂	♀	♂	♀	♂	♀	♂	♀	♂	♀	♂♀
LUTZOMYIINA	1	—	—	—	—	—	4	1	—	—	—	—	—	—	5 (0.92%)	1 (0.18%)	6 (1.10%)
*Brumptomyia* spp.	—	1	—	—	—	—	—	—	—	—	—	—	—	4	0	5 (0.92%)	5 (0.92%)
*Br*. *avellari*	9	9	—	—	5	2	—	—	—	—	—	—	12	7	26 (4.79%)	18 (3.31%)	44 (8.10%)
*Br*. *brumpti*	—	1	—	—	—	—	—	—	—	—	—	—	3	8	3 (0.55%)	9 (1.66%)	12 (2.21%)
*Evandromyia* spp.	—	—	—	—	—	—	—	2	—	—	—	—	—	—	0	2 (0.37%)	2 (0.37%)
*Ev*. (*Ald*.) *carmelinoi*	30	21	—	1	—	1	14	12	—	—	—	—	3	6	47 (8.66%)	41 (7.55%)	88 (16.21%)
*Ev*. (*Ald*.) *lenti*	7	5	—	—	—	—	8	7	—	—	—	—	—	2	15 (2.76%)	14 (2.58%)	29 (5.34%)
*Ev*. (*Ald*.) *termitophila*	—	—	—	—	—	—	1	—	—	—	—	—	—	—	1 (0.18%)	0	1 (0.18%)
*Ev*. (*Bar*.) *cortelezzii*	3	11	—	—	2	1	1	5	—	—	—	—	—	—	6 (1.10%)	17 (3.13%)	23 (4.24%)
*Ev*. (*Bar*.) *cortelezzii* complex	—	—	—	—	—	—	—	—	—	—	—	—	—	1	0	1 (0.18%)	1 (0.18%)
*Lu*. (*Lu*.) *longipalpis*	59	43	1	4	2	1	91	41	—	—	—	1	3	—	156 (28.73%)	90 (16.57%)	246 (45.30%)
*Nyssomyia* spp.	1	2	—	—	—	—	—	1	—	—	—	—	—	—	1 (0.18%)	3 (0.55%)	4 (0.74%)
*Ny*. *neivai*	2	6	—	—	3	3	4	—	—	—	—	—	—	—	9 (1.66%)	9 (1.66%)	18 (3.31%)
*Ny*. *whitmani*	36	8	—	1	2	—	4	—	—	—	—	—	1	1	43 (7.92%)	10 (1.84%)	53 (9.76%)
*Pa*. (*For*.) *brasiliensis*	—	—	—	—	1	1	1	—	—	—	—	—	—	—	2 (0.37%)	1 (0.18%)	3 (0.55%)
*Pa*. (*Xip*.) *hermanlenti*	—	—	—	—	—	—	—	—	—	—	—	—	—	1	0	1 (0.18%)	1 (0.18%)
*Sc. sordellii*	—	—	—	—	1	2	1	3	—	—	—	—	—	—	2 (0.37%)	5 (0.92%)	7 (1.29%)

Total	148	107	1	6	16	11	129	72	0	0	0	1	22	30	316 (58.19%)	227 (41.80%)	543 (100%)

## Data Availability

The data used to support the findings of this study are available from the corresponding author upon request.

## References

[1] Alvar J., Vélez I.D., Bern C. (2012). Leishmaniasis worldwide and global estimates of its incidence. *PLoS One*.

[2] Akhoundi M., Downing T., Votýpka J. (2017). *Leishmania* infections: molecular targets and diagnosis. *Molecular Aspects of Medicine*.

[3] Alvar J., Yactayo S., Bern C. (2006). Leishmaniasis and poverty. *Trends in Parasitology*.

[4] Saraiva L., Carvalho G. M. L., Gontijo C. M. F. (2009). Natural infection of *Lutzomyia neivai* and *Lutzomyia sallesi* (Diptera: Psychodidae) by *Leishmania infantum chagasi* in Brazil. *Journal of Medical Entomology*.

[5] Shaw J. J., Lainson R. (1972). Observations on the seasonal variations of *Lutzomyia flaviscutellata* in different types of forest and its relationship to enzootic rodent leishmaniasis (*Leishmania mexicana amazonensis*). *Transactions of the Royal Society of Tropical Medicine and Hygiene*.

[6] Maroli M., Feliciangeli M. D., Bichaud L., Charrel R. N., Gradoni L. (2013). Phlebotomine sandflies and the spreading of leishmaniases and other diseases of public health concern. *Medical and Veterinary Entomology*.

[7] Galati E. A. B., Rodrigues B. L. (2023). A review of historical phlebotominae taxonomy (Diptera: Psychodidae). *Neotropical Entomology*.

[8] Galati E. A. B. (2023). Morfologia e terminologia de Phlebotominae (Diptera: Psychodidae): Classificação e identificação de táxons das Américas, São Paulo: Apostila da disciplina de Bioecologia e Identificação de Phlebotominae. *Programa de Pós-Graduação em Saúde Pública. Faculdade de Saúde Pública/Universidade de São Paulo*.

[9] Barata R. A., França-Silva J. C., Mayrink W. (2005). Aspectos da ecologia e do comportamento de flebotomíneos em área endêmica de leishmaniose visceral Minas Gerais. *Revista da Sociedade Brasileira de Medicina Tropical*.

[10] de Dias F. O. P., Lorosa E. S., Rebêlo J. M. M. (2003). Fonte alimentar sangüínea e a peridomiciliação de *Lutzomyia longipalpis* (Lutz & Neiva, 1912) (Psychodidae, Phlebotominae). *Cadernos de Saude Publica*.

[11] Baum M., de Castro E. A., Pinto M. C. (2015). Molecular detection of the blood meal source of sand flies (Diptera: Psychodidae) in a transmission area of American cutaneous leishmaniasis, Paraná State, Brazil. *Acta Tropica*.

[12] Michalsky É.M., Fortes-Dias C. L., Pimenta P. F. P., Secundino N. F. C., Dias E. S. (2002). Assessment of PCR in the detection of *Leishmania* spp in experimentally infected individual phlebotomine sandflies (Diptera: Psychodidae: Phlebotominae). *Revista do Instituto de Medicina Tropical de São Paulo*.

[13] Paternina L. E., Verbel-Vergara D., Romero-Ricardo L. (2016). Evidence for anthropophily in five species of phlebotomine sand flies (Diptera: Psychodidae) from northern Colombia, revealed by molecular identification of bloodmeals. *Acta Tropica*.

[14] Killick-Kendrick R. (1999). The biology and control of phlebotomine sand flies. *Clinics in Dermatology*.

[15] Killick-Kendrick R. (1990). Phlebotomine vectors of the leishmaniases: a review. *Medical and Veterinary Entomology*.

[16] Alexander B., Maroli M. (2003). Control of phlebotomine sandflies. *Medical and Veterinary Entomology*.

[17] Abbasi I., Cunio R., Warburg A. (2009). Identification of blood meals imbibed by phlebotomine sand flies using cytochrome b PCR and reverse line blotting. *Vector-Borne Zoonotic Diseases*.

[18] Haouas N., Pesson B., Boudabous R., Dedet J.-P., Babba H., Ravel C. (2007). Development of a molecular tool for the identification of *Leishmania* reservoir hosts by blood meal analysis in the insect vectors. *The American Journal of Tropical Medicine and Hygiene*.

[19] Pan American Health Organization (2022). Leishmaniases: epidemiological report on the region of the Americas.

[20] Centro de Vigilância Epidemiológica “Prof. Alexandre Vranjac” (2023). Casos autóctones de Leishmaniose Tegumentar segundo GVE/município de infecção e ano de notificação, São Paulo, 2007 a 2022. *Sistema de Informação de Agravos de Notificação*.

[21] Centro de Vigilância Epidemiológica “Prof. Alexandre Vranjac” (2023). Casos confirmados de leishmaniose visceral segundo LPI e ano de notificaçã, Estado de São Paulo, 2017 a 2023. *Sistema de Informação de Agravos de Notificação*.

[22] Climate-Data.org (2022). Dados climáticos para cidades mundiais.

[23] Galvis-Ovallos F., Casanova C., da Sevá A. P., Galati E. A. B. (2017). Ecological parameters of the (S)-9-methylgermacrene-B population of the *Lutzomyia longipalpis* complex in a visceral leishmaniasis area in São Paulo state, Brazil. *Parasit Vectors*.

[24] Leonel J. A. F., Vioti G., Alves M. L. (2020). DNA extraction from individual Phlebotomine sand flies (Diptera: Psychodidae: Phlebotominae) specimens: Which is the method with better results?. *Experimental Parasitology*.

[25] Galati E. A. B., Rangel E. F., Shaw J. J. (2018). *Phlebotominae (Diptera, Psychodidae): Classification, Morphology and Terminology of Adults and Identification of American Taxa*.

[26] Marcondes C. B. (2007). A proposal of generic and subgeneric abbreviations for phlebotomine sandflies (Diptera: Psychodidae: Phlebotominae) of the world. *Entomological News*.

[27] Carvalho G. M. L., Rêgo F. D., Tanure A. (2017). Bloodmeal identification in field-collected sand flies from Casa Branca, Brazil, using the cytochrome b PCR method. *Journal of Medical Entomology*.

[28] Bruford M. W., Hanotte O., Brookfield J. F. Y., Burke T., Hoelzel A. R. (1998). *Multilocus and Singlelocus DNA Fingerprinting, Molecular Genetic Analysis of Populations: A Practical Approach*.

[29] Lins R. M. M. A., Oliveira S. G., Souza N. A. (2002). Molecular evolution of the cacophony IVS6 region in sandflies. *Insect Molecular Biology*.

[30] Pita-Pereira D. D., Alves C. R., Souza M. B. (2005). Identification of naturally infected *Lutzomyia intermedia* and *Lutzomyia migonei* with *Leishmania (Viannia) braziliensis* in Rio de Janeiro (Brazil) revealed by a PCR multiplex non-isotopic hybridisation assay. *Transactions of the Royal Society of Tropical Medicine and Hygiene*.

[31] Volpini A. C., Passos V. M. A., Oliveira G. C., Romanha A. J. (2004). PCR-RFLP to identify *Leishmania* (*Viannia*) *braziliensis* and *L*. (*Leishmania*) *amazonensis* causing American cutaneous leishmaniasis. *Acta Tropica*.

[32] El Tai N. O., Osman O. F., El Fari M., Presber W., Schönian G. (2000). Genetic heterogeneity of ribosomal internal transcribed spacer in clinical samples of *Leishmania donovani* spotted on filter paper as revealed by single-strand conformation polymorphisms and sequencing. *Transactions of the Royal Society of Tropical Medicine and Hygiene*.

[33] Steuber S., Abdel-Rady A., Clausen P. H. (2005). PCR-RFLP analysis: a promising technique for host species identification of blood meals from tsetse flies (Diptera: Glossinidae). *Zeitschrift Fur Parasitenkunde-Parasitology Research*.

[34] Carvalho G. M. L., Brazil R. P., Falcão A. L., Andrade-Filho J. D. (2009). Distribuição geográfica do Complexo *cortelezzii* (Diptera: Psychodidae: Phlebotominae) no Brasil. *Neotropical Entomology*.

[35] Cardim M. F. M., Guirado M. M., Dibo M. R., Chiaravalloti-Neto F. (2016). Leishmaniose visceral no estado de São Paulo, Brasil: análise espacial e espaço-temporal. *Rev Saude Publica*.

[36] Ministério da Saúde (2014). *Manual de Vigilância e Controle da Leishmaniose Visceral*.

[37] Ministério da Saúde (2017). Manual de Vigilância da Leishmaniose Tegumentar.

[38] Rêgo F. D., Soares R. P. (2021). *Lutzomyia longipalpis*: an update on this sand fly vector. *Anais da Academia Brasileira de Ciencias*.

[39] Carvalho G. M. L., Andrade-Filho J. D., Falcão A. L., Lima A. C. V. M. R., Gontijo C. M. F. (2008). Naturally infected *Lutzomyia* sand flies in a *Leishmania*-endemic area of Brazil. *Vector-Borne Zoonotic Diseases*.

[40] Lana R. S., Michalsky É.M., Fortes-Dias C. L. (2015). Phlebotomine sand fly fauna and *Leishmania* infection in the vicinity of the Serra do Cipó National Park, a natural brazilian heritage site. *Biomed Research International*.

[41] Bezerra-Vasconcelos D. R., Melo L. M., Albuquerque É.S., Luciano M. C. S., Bevilaqua C. M. L. (2011). Real-time PCR to assess the *Leishmania* load in *Lutzomyia longipalpis* sand flies: screening of target genes and assessment of quantitative methods. *Experimental Parasitology*.

[42] González E., Álvarez A., Ruiz S., Molina R., Jiménez M. (2017). Detection of high *Leishmania infantum* loads in *Phlebotomus perniciosus* captured in the leishmaniasis focus of southwestern Madrid region (Spain) by real time PCR. *Acta Tropica*.

[43] Rossi E., Bongiorno G., Ciolli E. (2008). Seasonal phenology, host-blood feeding preferences and natural *Leishmania* infection of *Phlebotomus perniciosus* (Diptera, Psychodidae) in a high-endemic focus of canine leishmaniasis in Rome province, Italy. *Acta Tropica*.

[44] Silva R. C. R., Cruz L. N. P. D., Coutinho J. M. S., Fonseca-Alves C. E., Rebêlo J. M. M., Pereira S. R. F. (2021). Experimental transmission of Leishmania (Leishmania) amazonensis to immunosuppressed mice through the bite of Lutzomyia longipalpis (Diptera: Psychodidae) results in cutaneous leishmaniasis. *Revista do Instituto de Medicina Tropical de Sao Paulo*.

[45] Carvalho-Silva R., Ribeiro-da-Silva R. C., Cruz L. N. P. D. (2022). Predominance of Leishmania (Leishmania) amazonensis DNA in Lutzomyia longipalpis sand flies (Diptera: Psychodidae) from an endemic area for leishmaniasis in Northeastern Brazil. *Revista do Instituto de Medicina Tropical de Sao Paulo*.

[46] Pinheiro M. P. G., Silva-Inacio C. L., de Mederios Silva M. M., Sergio P., de Araujo F., de Ximenes M. F. F. (2021). Potential vectors of *Leishmania* spp. in an Atlantic Forest conservation unit in northeastern Brazil under anthropic pressure. *Parasite & Vectors*.

[47] Bravo-Barriga D., Parreira R., Maia C. (2016). Detection of *Leishmania* DNA and blood meal sources in phlebotomine sand flies (Diptera: Psychodidae) in western of Spain: update on distribution and risk factors associated. *Acta Tropica*.

[48] da Costa G. S., Júnior A. M. P., Castro T. S., de Ferreira P.F.M. P. G. E. M., Medeiros J. F. (2021). Sand fly fauna and molecular detection of *Leishmania* species and blood meal sources in different rural environments in western Amazon. *Acta Tropica*.

[49] de Avila M. M., Brilhante A. F., de Souza C. F., Bevilacqua P. D., Galati E. A. B., Brazil R. P. (2018). Ecology, feeding and natural infection by Leishmania spp. of phlebotomine sand flies in an area of high incidence of American tegumentary leishmaniasis in the municipality of Rio Branco, Acre, Brazil. *Parasite & Vectors*.

[50] Teodoro U., La Salvia-Filho V., de L. E. M. (1993). Flebotomíneos em área de transmissão de leishmaniose tegumentar na região norte do Estado do Paraná - Brasil: Variação sazonal e atividade noturna. *Rev Saude Publica*.

[51] Quinnell R. J., Courtenay O. (2009). Transmission, reservoir hosts and control of zoonotic visceral leishmaniasis. *Parasitology*.

[52] Roque A. L. R., Jansen A. M. (2014). Wild and synanthropic reservoirs of *Leishmania* species in the Americas. *International Journal for Parasitology. Parasites and Wildlife*.

[53] Carranza-Tamayo C. O., Werneck G. L., Romero G. A. S. (2016). Are opossums a relevant factor associated with asymptomatic *Leishmania* infection in the outskirts of the largest Brazilian cities?. *The Brazilian Journal of Infection Diseases*.

[54] Guimarães-e-Silva A. S., de Silva S. O., da S. R. C. R., Pinheiro V. C. S., Rebêlo J. M. M., Melo M. N. (2017). *Leishmania* infection and blood food sources of phlebotomines in an area of Brazil endemic for visceral and tegumentary leishmaniasis. *PLoS One*.

[55] Vioti G., Leonel J. A. F., Lemes K. M. (2019). Molecular detection of *Leishmania* spp. in cattle from Brazil by means of PCR using internal transcribed spacer 1. *Brazilian Journal of Veterinary Parasitology*.

[56] de Souza C. F., Quaresma P. F., Filho J. D. A., Bevilacqua P. D. (2014). Phlebotomine fauna in the urban area of Timóteo, State of Minas Gerais, Brazil. *Acta Tropica*.

[57] Tolezano J. E., Taniguchi H. H., Elias C. R., Larosa R. (2001). Epidemiologia da Leishmaniose Tegumentar Americana (LTA) no Estado de São Paulo. III. Influência da ação antrópica na sucessão vetorial da LTA. *Revista do Instituto Adolfo Lutz*.

[58] Shimabukuro P. H. F., Galati E. A. B. (2011). Lista de espécies de Phlebotominae (Diptera, Psychodidae) do Estado de São Paulo, Brasil, com comentários sobre sua distribuição geográfica. *Biota Neotropica*.

